# Glucose tolerance in mice exposed to light–dark stimulus patterns mirroring dayshift and rotating shift schedules

**DOI:** 10.1038/srep40661

**Published:** 2017-01-12

**Authors:** Mariana G. Figueiro, Leora Radetsky, Barbara Plitnick, Mark S. Rea

**Affiliations:** 1 Lighting Research Center, Rensselaer Polytechnic Institute, Troy, NY, USA

## Abstract

Glucose tolerance was measured in (nocturnal) mice exposed to light–dark stimulus patterns simulating those that (diurnal) humans would experience while working dayshift (DSS) and 2 rotating night shift patterns (1 rotating night shift per week [RSS1] and 3 rotating night shifts per week [RSS3]). Oral glucose tolerance tests were administered at the same time and light phase during the third week of each experimental session. In contrast to the RSS1 and RSS3 conditions, glucose levels reduced more quickly for the DSS condition. Glucose area-under-the-curve measured for the DSS condition was also significantly less than that for the RSS1 and RSS3 conditions. Circadian disruption for the 3 light–dark patterns was quantified using phasor magnitude based on the 24-h light–dark patterns and their associated activity–rest patterns. Circadian disruption for mice in the DSS condition was significantly less than that for the RSS1 and RSS3 conditions. This study extends previous studies showing that even 1 night of shift work decreases glucose tolerance and that circadian disruption is linked to glucose tolerance in mice.

The natural 24-h light–dark cycle incident on mammalian retinae is the primary synchronizer of cellular, physiological, and behavioral rhythms to local position on Earth^1^,^2^. Electrical signals emanating from retinal neurons are carried over the retinohypothalamic tract (RHT) to the master biological clock in the suprachiasmatic nuclei (SCN), which plays a key role in the timing of biological systems ranging from mitotic cell division^3^ to endocrine synthesis^4^ to behavioral sleep^5^. Deviations from a regular, 24-h light–dark pattern, such as those that occur with rotating shift work or rapid trans-meridian flight, can compromise the functionalities of rhythmic biological systems. The term “circadian disruption” has been coined to encompass a wide range of acute and chronic decrements in performance, sleep, wellbeing, and health that are associated with irregular exposures to light and dark^6^,^7^,^8^,^9^,^10^,^11^,^12^,^13^.

It is more practical and less expensive to use animal models rather than humans to perform parametric studies of light-induced circadian disruption and its possible effects on health outcomes. To increase face validity, a functional bridge must be built between exposures to irregular light–dark stimulus patterns that are actually experienced by humans and simulated, parametrically controlled light–dark stimulus patterns in animal models. Since most animal models are nocturnal rodents, this functional bridge must also consider species differences in the spectral and absolute sensitivities to light.

To quantify circadian disruption in humans and animal models, Rea *et al*. proposed the use of phasor analysis^14^. Phasor analysis has been used to quantify the synchrony between 24-h light–dark stimulus and daily activity–rest response patterns^14^. This analysis yields a vector called a phasor, which quantifies how well these patterns are synchronized over a 24-h period (i.e. phasor magnitude) and their stimulus–response phase relationship (i.e. phasor angle).

It is perhaps worth emphasizing a key insight gained from the development of phasor analysis—namely, that measurements of light exposures *per se*, even species-specific light–dark exposures, are not helpful for understanding circadian entrainment and disruption. Rather, as the stimulus for circadian entrainment is the 24-h *pattern* of light–dark exposures, measurements must be made over several days to quantitatively bridge circadian disruption in diurnal humans to nocturnal mice, and vice versa. Previous research has shown that light–dark stimulus patterns measured using calibrated personal light-measurement devices worn by humans in the field can be translated into calibrated, controlled light–dark stimulus patterns for implementation in mouse cages^15^. These patterns as they affect circadian disruption and health outcomes can then, in turn, be measured in the mouse model.

In the present study, we exposed mice to light–dark patterns simulating the measured light–dark patterns experienced by dayshift and rotating shift workers. We then measured how those patterns differentially affected circadian disruption (i.e. phasor analysis) and a health outcome (i.e. glucose tolerance) in a mouse model. Specifically, we investigated how glucose tolerance was affected by exposure to light–dark stimulus patterns during 3 experimental conditions: (1) simulated dayshift (DSS), (2) simulated rotating shift work including 1 night per week (RSS1), and (3) simulated rotating shift work including 3 nights per week (RSS3). Circadian disruption was quantified via the calculation of phasor magnitudes for mouse-specific^16^ 24-h light–dark stimulus and 24-h activity–rest (i.e. wheel-running) patterns.

As a previous study^15^ showed similar phasor magnitudes for humans and mice exposed to similar, species-specific light–dark stimulus patterns, it was hypothesized that glucose tolerance would be better for the DSS condition than for the RSS1 and RSS3 conditions. Moreover, it was expected that phasor magnitudes measured from mice would correlate with measures of glucose tolerance. The present study confirmed results from previous studies showing that circadian disruption, similar to that experienced by shift workers, decreases phasor magnitude in mice^15^ while also extending those results to suggest that phasor magnitude, a measure of circadian entrainment, is correlated with glucose tolerance. The present study also supports the inference that phasor magnitudes measured from humans living their normal lives can be translated into parametric studies of circadian disruption in animal models to estimate health risks, such as Type II diabetes.

## Materials and Methods

Twenty-four C57BL/6 male mice (obtained from Taconic Biosciences, Inc., Hudson, NY), approximately 12 weeks old at the start of the experiment, were individually housed in a dedicated facility in the Rensselaer Polytechnic Institute BioResearch Core. Animals arrived in the facility when they were 8 weeks old, and placed in the experimental room on a 12-h light:12-h dark (12L:12D) lighting condition when they were 10 weeks old. Rensselaer Polytechnic Institute’s Institute Animal Care and Use Committee (IACUC) approved the study, and our experiment was performed in accordance with relevant guidelines and regulations. All animal studies conducted by our research team (Lighting Research Center, Rensselaer Polytechnic Institute) conform to international ethical standards^17^. Food (Prolab Isopro RMH 3000 irradiated chow [LabDiet, St. Louis, MO]) and sterile water were available *ad libitum* for the duration of the experiment. The cages were located in a ventilated rack in a light-tight room. Access to the cage room from the corridor was through a dark anteroom. Sweeps on the bottom of the anteroom doors prevented stray light from entering the cage room. The animals were monitored once per day at variable times between 10:00 AM and 3:00 PM on weekdays and between 7:00 AM and 7:00 PM on weekends.

The 24 animals were evenly divided into 2 groups, and all animals were individually caged but simultaneously received the same experimental lighting conditions because they were housed in the same room. One group (n = 12) was placed in cages equipped with running wheels connected to a wheel monitoring system (VitalView, Philips Respironics, Pittsburgh, PA), and phasor analysis was used to quantify circadian disruption from the recorded activity–rest and light–dark data. No glucose tolerance testing was administered to this group. The other group (n = 12) did not have access to running wheels, and blood samples were drawn from these animals for glucose tolerance testing. Activity–rest patterns for this latter group of animals could not be measured by other means (e.g. infrared sensors), because the ventilated cage racks housing the animals would not accommodate such devices. As one of the animals in the second group had to be euthanized during the last experimental session, only the glucose tolerance results for the 11 animals completing the study are reported here.

### Lighting

A cage-lighting system was developed and installed for the experiment. The spectral and absolute sensitivities of the murine circadian system determined by Bullough *et al*.^16^. were used to set the spectral power distribution of the light provided to the cages. Diffuse illumination was provided for every cage interior by custom light fixtures placed on both sides of the transparent cage walls. Each light fixture contained 2 green light-emitting diodes ([LEDs] peak wavelength = 519 nm, full-width half-maximum [FWHM] bandwidth = 40 nm) under PTFE (Teflon) diffusers. A DMX system (iPlayer 3 controller and ColorPlay 3 software, Philips Color Kinetics, Burlington, MA) was used to provide 4 μW/cm^2^ at the center of each cage floor and to produce the experimental light–dark conditions. The cage-lighting system design was based on our research into the circadian phototransduction mechanisms of humans and mice. We showed that the spectral sensitivity of the mouse circadian phototransduction mechanism is greatest to green light and the absolute sensitivity to light is between 3,000 and 10,000 times greater than it is for humans. Thus, at 4 μW/cm^2^ of 519 nm light, the cage-lighting system provided a light stimulus comparable to that experienced by humans in the built environment^16^,^18^.

With the exception of a few indicator LED lights on the equipment and the digital display for the cage rack, no other lights were energized in the cage room. The lights and recessed fluorescent lighting in the connected anteroom were always covered with bandpass filters blocking light emission shorter than 600 nm (Rosco 27 [Rosco Laboratories, Stamford, CT] and Lee 6 [Lee Filters, Burbank, CA]). Technicians who cared for the animals used the same type of bandpass filters to cover their flashlight lenses. Thus, the only biologically meaningful light for the mice in the experiment was provided by the custom light fixtures placed adjacent to the cage walls.

### Experimental Conditions

**Figure 1:**
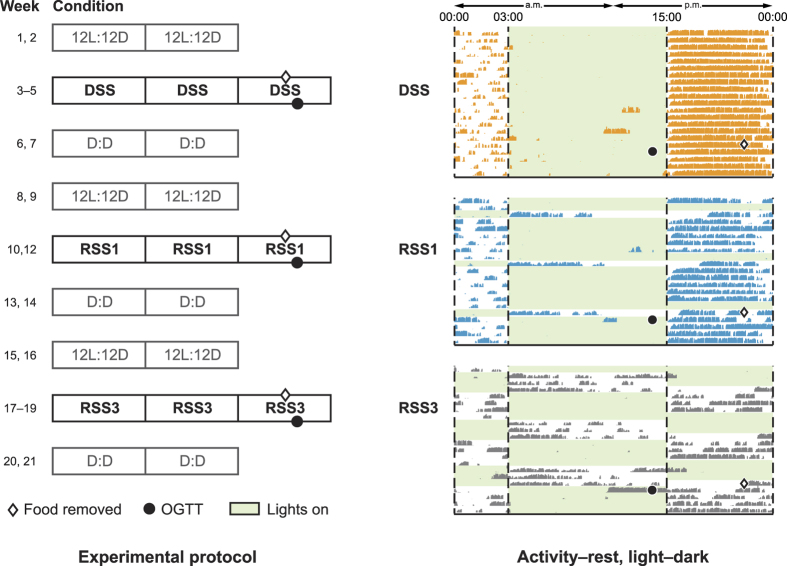
Experimental protocol for light–dark stimulus and oral glucose tolerance test (OGTT) measurements (left), along with an example of light–dark stimulus and recorded activity–rest patterns (right). The green shading indicates when the LED lighting was switched on. Food was removed from the cages 14 h before the OGTT. The actigraphy plot (right) shows the results recorded for a single animal (mouse/cage 21) through all 3 experimental sessions (labeled in boldface). The wheel-running mice did not undergo OGTT, but the times of food removal and OGTT are indicated to show their relationship to the administered light–dark stimulus patterns.

Prior to the first experimental session, all animals were exposed to a 12 L:12D lighting condition. All 24 animals were then exposed, in turn, to 3 experimental conditions: (1) a 12 L:12D pattern simulating a dayshift schedule (DSS); (2) a 12L:12D pattern with 1 simulated night of rotating shift work per week (RSS1); and (3) a 12 L:12D pattern with 3 consecutive simulated nights of rotating shift work per week (RSS3). The simulated rotating shift conditions were comprised of 1 (RSS1) or 3 (RSS3) counterphased 12D:12 L patterns. The animals were exposed to the 3 experimental conditions in 3 sessions that each lasted 3 consecutive weeks. Following each experimental condition, the animals were placed in continuous darkness (D:D) for 2 weeks and then exposed to a 12 L:12D condition for a minimum of 2 weeks before starting the next experimental session. Tau D:D was calculated after each experimental condition to determine the carryover effects from the preceding experimental condition. As some carryover effect was expected, the 2 weeks of 12 L:12D lighting conditons prior to starting a new experimental condition served as a washout period. The entire protocol lasted 21 weeks (Fig. 1).

### Glucose Protocol

Oral glucose tolerance tests (OGTTs) were administered at the same time–light phase during the third week of each experimental session, exactly 3 h before the start of the dark phase (see Fig. 1). The timing of the OGTT was chosen to correspond to a point near the peak of the daily glucose rhythm in C57BL/6 mice^19^. The 12 animals were handled twice prior to each test to acclimate them to the procedure, usually before the lights were switched off. For all 3 experimental conditions, the veterinarian technician repeated the same procedure of holding the animal in her hands and collecting blood from its tail vein.

At 14 h prior to the OGTT for all 3 experimental conditions, food was removed from the cages. The duration of this fasting period was selected to ensure that our findings would be comparable with others in the literature, based on the observation that 73 out of the 100 studies surveyed by Andrikopoulos *et al*.^20^. fasted animals for 14 h or longer. The researcher entered the cage room at 10:00 PM in darkness (using a red spectrum flashlight), removed the cage from the rack, and placed it on the bench. The cage lid was removed, all food present in the food container was removed and disposed of, and the cage was placed back in the rack. Water was provided, and the animal was not handled. All food was removed from all cages in this manner in less than 10 min.

The fasting animals were weighed and the initial tail snip was performed 2 h prior to obtaining the first glucose measurement. The glucose dosage for each animal was calculated at 2 g/kg following a standard approach. The conscious animals were orally gavaged and glucose levels were assessed by collecting 0.3 μL of blood immediately before (T0) and subsequently at 15 min (T1), 30 min (T2), 60 min (T3), and 120 min (T4) after glucose administration. Blood glucose was measured using an AlphaTRAK whole-blood glucose monitor (Abbott Laboratories, Abbott Park, IL).

### Data Analyses

Glucose levels measured at each time point (T0–T4) were employed for a 3 (lighting patterns) × 5 (measurement times) analysis of variance (ANOVA). *Post hoc* two-tailed, Student’s t-tests were used to determine whether the glucose levels at each measurement time were significantly different between the 3 experimental conditions. In all statistical analyses, adjustments for multiple comparisons were performed using the Sidak method^21^,^22^.

The absolute glucose levels measured over each 120-min OGTT were also used to calculate glucose area-under-the-curve (AUC) values using the trapezoidal numerical integration method, also referred to as the trapezoidal rule^20^,^23^. One-way repeated measures ANOVA and Student’s t-tests were used to assess whether there was a significant change in glucose AUC between the DSS condition and the RSS1 and RSS3 experimental conditions.

Phasor analysis, a technique based on signal processing techniques, was employed to interpret the relationship between the experimental light–dark stimulus conditions and the activity–rest data recorded for the 12 animals provided with running wheels. A detailed description of this technique is provided in research published by Miller *et al*.^24^. In brief, the synchrony between the periodic changes in light–dark and activity–rest were first determined by calculating the circular correlation function of the light and activity time series recorded over the 3-week period of each experimental condition. The circular correlation function was then decomposed into its temporal frequencies and phase angles using Fourier analysis techniques, from which the 24-h frequency component was selected as a measure of circadian rhythmicity. The 24-h phasor magnitude was used as the metric for circadian entrainment/disruption; the greater the magnitude, the greater the level of circadian entrainment of activity to light. One-way repeated measures ANOVA and Student’s t-tests were used to assess whether there was a significant change in phasor magnitudes and tau D:D between the DSS condition and the RSS1 and RSS3 experimental conditions.

Two software programs were used to analyze the wheel-running data. VitalView (STARR Life Sciences, Oakmont, PA) was used to collect the data and create actogram (AWD) files, and MATLAB (MathWorks, Natick, MA) was used to calculate phasor magnitudes and tau D:D after each experimental condition.

## Results

### Glucose Measurements

**Figure 2:**
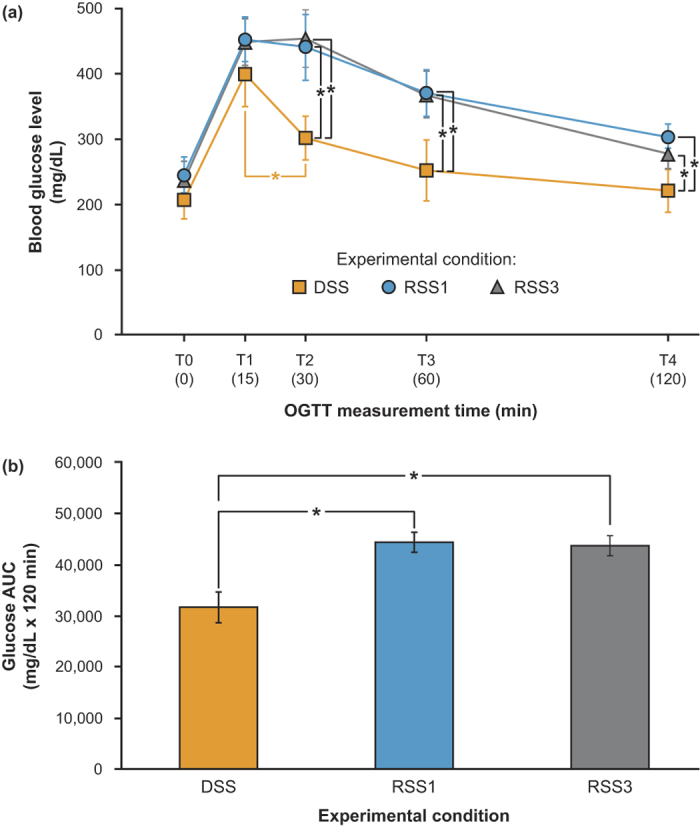
Means ± SD of glucose levels measured at each of the measurement times, and (**b**) mean ± SD of the glucose area-under-the-curve (AUC) for the 3 lighting patterns. Blood glucose levels and glucose area under the curve with AUC were significantly higher following OGTT administration after animals experienced both the RSS1 and RSS3 conditions, compared to experiencing the DSS condition, suggesting that glucose tolerance is significantly decreased after exposing animals to light–dark stimulus patterns simulating those experienced by rotating shift workers who work both 1 night and 3 nights per week (*statistically significant).

The one-way repeated measures ANOVA performed on the glucose measurements revealed a significant main effect of lighting patterns (*F*_*2,20*_ = 117.7, *p* < 0.0001), a significant main effect of measurement times (*F*_*4,40*_ = 210.6, *p* < 0.0001), and a significant lighting patterns × measurement times interaction (*F*_*8,80*_ = 7.1, *p* < 0.0001). Student’s t-tests revealed significantly lower glucose levels for the DSS condition compared to the RSS1 (*t*_*10*_ = 14.8, *p* < 0.0001) and RSS3 (*t*_*10*_ = 14.7, *p* < 0.0001) conditions. Glucose levels were significantly higher for the RSS1 and RSS3 conditions at all measurement times. Student’s t-tests revealed that glucose levels were significantly lower for the DSS condition compared to the RSS1 condition at T0 (*t*_*10*_ = 3.1, *p* = 0.01), at T1 (*t*_*10*_ = 3.1, *p* = 0.01), at T2 (*T*_*10*_ = 7.9, *p* < 0.0001), at T3 (*t*_*10*_ = 8.2, *p* < 0.0001), and at T4 (*t*_*10*_ = 7.6, *p* < 0.0001). Glucose levels were also significantly lower for the DSS condition compared to the RSS3 condition at T0 (*t*_*10*_ = 2.3, *p* = 0.046), at T1 (*t*_*10*_ = 3.5, *p* = 0.006), at T2 (*t*_*10*_ = 7.9, *p* < 0.0001), at T3 (*t*_*10*_ = 6.8, *p* < 0.0001), and at T4 (*t*_*10*_ = 4.8, *p* = 0.001). The mean ± standard deviation (SD) glucose levels at the OGTT measurement times for the 3 experimental conditions are shown in Fig. 2a.

### Glucose AUC

The ANOVA performed on the glucose AUC values revealed a significant main effect of lighting patterns (*F*_*2,20*_ = 114.6, *p* < 0.0001). Paired two-tailed Student’s t-tests revealed that glucose AUC measured for the DSS condition was significantly less than that measured for the RSS1 and RSS3 conditions (*t*_*10*_ = 13.8, *p* < 0.0001; *t*_*10*_ = 11.8, *p* < 0.0001; respectively). No significant difference was identified between the glucose AUC values for the RSS1 and RSS3 conditions (*t*_*10*_ = 0.76, *p* = 0.47). The mean ± SD AUC for all 3 experimental conditions is shown in Fig. 2b.

### Phasor Magnitude and tau D:D after each experimental condition

**Figure 3:**
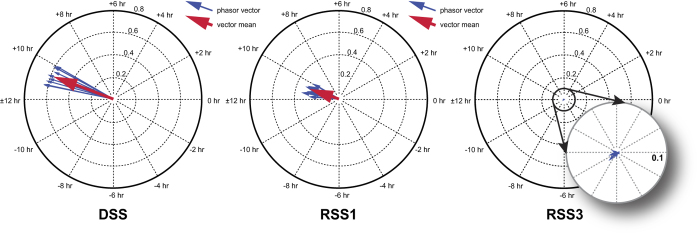
Phasor magnitudes for the DSS, RSS1, and RSS3 lighting patterns. Phasor magnitudes calculated from animals experiencing the DDS condition were significantly greater than those calculated from animals experiencing the RSS1 and RSS3 conditions, suggesting greater circadian behavioral entrainment in animals experiencing light-dark stimulus patterns simulating dayshift workers than in animals experiencing light-dark patterns simulating rotating shift workers who work both 1 night and 3 nights per week.

The ANOVA revealed a significant main effect of lighting patterns (F_*2,22*_ = 547; *p* < 0.0001). Phasor magnitudes measured for the DSS condition were significantly greater than those measured for the RSS1 and RSS3 conditions (t_*11*_ = 20.6, *p* < 0.0001; t_*11*_ = 24.7; *p* < 0.0001; respectively). Phasor magnitude was also significantly greater for the RSS1 condition than for the RSS3 condition (t_*11*_ = 22.1, *p* < 0.0001). The mean ± SD phasor magnitude was 0.58 ± 0.08 for the DSS condition, 0.28 ± 0.04 for the RSS1 condition, and 0.008 ± 0.005 for the RSS3 condition. The phasor magnitudes for the DSS, RSS1, and RSS3 conditions are shown in Fig. 3.

The ANOVA revealed a significant main effect of lighting patterns (*F*_*2,33*_ = 5.40, *p* = 0.009). Tau D:D following the RSS1 condition was significantly shorter than following the DSS (*t*_*12*_ = 3.69, *p* = 0.004) and RSS3 (*t*_*12*_ = 4.90, *p* = 0.0005) conditions. No other significant differences were observed. The mean ± SD tau D:D was 23.6 ± 0.16 following the DSS condition, 23.4 ± 0.21 following the RSS1 condition, and 23.5 ± 0.21 following the RSS3 condition.

## Discussion

Previous laboratory studies have shown that circadian disruption is associated with decreased glucose tolerance in both animals and humans^6^,^25^. The present study investigated how circadian disruption measured over the course of 3 consecutive weeks affected glucose tolerance in nocturnal mice. These results add experimental evidence to the literature showing that glucose tolerance is significantly decreased after exposing animals to light–dark stimulus patterns simulating those experienced by rotating shift workers who work both 1 night and 3 nights per week. The simulated rotating shift conditions (RSS1 and RSS3) also had significantly higher glucose AUC than the simulated dayshift (DSS) condition. The results of this study also suggest that even 1 night of shift work may increase the likelihood of diabetes relative to a consistent dayshift schedule, at least in the short term. A protocol similar to the one employed in the present study was used by Van Dycke *et al*.^26^, who showed that chronic circadian disruption resulting from weekly alternating light–dark cycles increased breast cancer development in p53(R270H/+) WAPCre conditional mutant mice. In addition, that study showed that an 18-week period of alternating light–dark cycles was also associated with an increase in body weight^26^. Future studies are needed to investigate whether the animals would adapt to an RSS1 condition better than to an RSS3 condition if they experienced these alternating conditions for more than 18 weeks.

The present study is unique because light level and spectrum in the animal cages were calibrated so that the mice, which are nocturnal animals and much more sensitive to light than humans^18^, received light–dark stimuli comparable to those measured by Daysimeters worn by dayshift and rotating-shift nurses in the field^24^. Consistent with a previous study demonstrating that phasor magnitudes provide a common, quantitative measure of circadian disruption for both diurnal humans and nocturnal animal models^15^, the present results suggest that there is a relationship between phasor magnitude and glucose tolerance. Personal light–dark and activity–rest patterns measured in the field have previously been used to calculate phasor magnitudes for people working dayshift and rotating shift schedules^24^. Similarly, phasor magnitudes can be calculated for laboratory animals experiencing controlled cage-lighting patterns and wheel-running behavior patterns. As shown previously^15^, the same light–dark stimulus patterns actually experienced by people working dayshift and rotating shift schedules can be replicated with mouse-specific, calibrated light–dark stimulus patterns, yielding nearly identical phasor magnitudes^27^.

In this study specifically, analysis of the relationship between glucose AUC measured for the experimental group and phasor magnitudes calculated for the wheel-running animals identified a significant negative correlation suggesting that as phasor magnitude increases (i.e., less circadian disruption), the glucose AUC is reduced. This relationship, in turn, suggests a greater clearance of glucose by the animal, or better glucose tolerance. It is important to point out, however, that this comparison was made using between-subjects data. This between-subjects protocol was used for several reasons: (1) exercise alone may affect metabolism (and, therefore, glucose tolerance), and we were mainly interested in investigating glucose levels alone rather than the interaction between glucose levels and exercise; (2) exercise may also affect the SCN’s activity and its outputs^28^,^29^; (3) logistically, only a single cage room in the animal research facility was available and outfitted with our special cage lighting system; and (4) there was no available physical space above the ventilated cage racks to install infrared sensors to measure activity–rest patterns. Future studies should measure glucose levels in animals on the wheels, so that a direct relationship in the same animals can be established.

A second limitation of the present study is the fact that the same animals experienced all 3 successive experimental conditions, and therefore, age may have played a role in the observed decrease in glucose tolerance between the DSS condition (the first condition that animals experienced) and the RSS1 and RSS3 conditions (the second and third conditions that animals experienced, respectively). Future studies should reverse the order of the experimental conditions or use a between-subjects experimental design to confirm these results. As mentioned above, for the present study we had access to only a single cage room, which would have hindered our ability to efficiently conduct the research with 3 separate groups of animals. It is important to note, however, that if age had played a major role in the results, the glucose levels for the RSS1 condition would have been significantly lower than those for the RSS3 condition, but this was not the case. Moreover, new unpublished data collected to test this order effect show that age-matched animals who experienced the DSS and the RSS3 as their first experimental conditions showed glucose levels similar to those presented here. That is, glucose tolerance was reduced 30 min, 60 min, and 120 min after glucose administration in the RSS3 condition compared to the DSS condition.

A third limitation of the present study is that we did not measure the animals’ food intake and determine whether the weight gain associated with the RSS1 and RSS3 conditions resulted from them eating at the wrong circadian time, as shown by Arble *et al*. and others^30^,^31^,^32^,^33^, or whether they were eating more while experiencing these lighting conditions.

A fourth possible limitation of our research is that the light–dark patterns for the 48-h period preceding the GTTs were not equivalent for the DSS (12 L:12D, 12 L:12D), RSS1 (12 L:12 L, 12D, 12D), and RSS3 (12D:12 L, 12D:12D) experimental conditions. It should be noted, however, that shift workers who work 3 consecutive shifts tend to remain awake after the final one to facilitate sleep the following evening^34^, so this light–dark schedule is representative of what a shift worker would experience in real life.

Despite these limitations, the present study represents a possible step toward the goal of more directly understanding the health implications of circadian disruption in humans by offering a possible quantitative bridge between (1) ecologically relevant light and activity data from shift workers and (2) parametric investigations of health outcomes from animal models. Future epidemiological studies investigating the effects of circadian disruption on human health should now be able to point the way to relevant, specific, and systematic investigations of the relationship between circadian disruption and health outcomes using animal models.

## Additional Information

**How to cite this article**: Figueiro, M. G. *et al*. Glucose tolerance in mice exposed to light–dark stimulus patterns mirroring dayshift and rotating shift schedules. *Sci. Rep.* **7**, 40661; doi: 10.1038/srep40661 (2017).

**Publisher's note:** Springer Nature remains neutral with regard to jurisdictional claims in published maps and institutional affiliations.
